# Electrical Impedance to Easily Discover Undeclared Freeze-thaw Cycles in Slaughtered Bovine Meat

**DOI:** 10.2478/joeb-2021-0002

**Published:** 2021-11-20

**Authors:** A. H. Dell’Osa, G. Battacone, G. Pulina, A. Fois, F. Tocco, A. Loviselli, A. Concu, F. Velluzzi

**Affiliations:** 1 Instituto de Desarrollo Económico e Innovación, Universidad Nacional de Tierra del Fuego, Ushuaia, Argentina; 2 Department of Agricultural Science, University of Sassari, Sassari, Italy; 3 Nomadyca Ltd, Kampala, Uganda; 4 Department of Medical Sciences and Public Health, University of Cagliari, Cagliari, Italy; 5 2C Technologies Ltd, Academic Spin-Off, University of Cagliari, Cagliari, Italy

**Keywords:** Meat anisotropy, slaughtered bovine meat, freeze-thaw cycles, meat electrical bioimpedance, meat maturation process

## Abstract

A portable electrical impedance spectroscopy device was developed to monitor the bioimpedance resistive component of bovine meat by injecting a sinusoidal current of 1 mA at 65 kHz. Both right and left longissimus dorsi muscles were trimmed from 4 slaughtered cows. The left muscle portions were frozen to −18 °C for 7 days while the right ones were meantime maintained at 5 °C. Mean value of impedance per length (Ω/cm) of frozen and thawed left samples was 31% lower than that of right non-frozen one (P = 0.0001). It was concluded that the device is reliable for monitoring the maturation of beef meat in situ with the possibility of revealing undeclared freeze-thaw cycles.

## Introduction

From a structural point of view, the trimmed meat from bovine muscle is an anisotropic tissue, which is characterized by a composite network of muscle fiber bundles containing aligned myofibers surrounded by a fine endomysial envelope of connective tissue. Several structural levels that characterize the muscle's morphology have highly contrasted electrical and dielectric properties. In fact, the meat's electrical properties result in part from non-frequency-dependent materials, which define the meat's resistance, i.e. an array of highly elongated structures with high longitudinal conductance due to both intra- and extra-cellular presence of ions, which are surrounded by connective sheaths with a very low conductance. Electrical characteristics of meat are also due to frequency-dependent materials, which define its capacitive reactance due to the cell membrane property of maintaining the separation of the negative from positive electrical charges across it, as in an electrical capacitor. Both these electrical specificities give rise to an electric anisotropy of the muscle, which is closely dependent on its histological characteristics.

In slaughtered meat, it has been found that during the post-rigor period (which begins 2 – 3 days after slaughter) the behavior of its electrical impedance (Z_m_) reflects major changes occurring in the meat's structure [1], and these modifications are related to the disruption of the myofibrillar organization of the cytoskeleton and of cell membranes, due to protease activity [2]. In fact, during this time-dependent proteolysis, i.e. the meat's maturation, degradation occurs in proteins with structural tears and myofibril fragmentation, together with degradation in cytoskeleton architecture [3]. These structural modifications of trimmed muscle give rise to a progressive loss of structural anisotropy from which a reduction of Z_m_ also occurs [4,5].

The optimization of the slaughtered meat maturation period, paying close attention that the maturation process takes place properly, is one of the main goals of the meat industry, particularly of the beef sector [6]. In fact, this production sector has to contend with the broad-ranging variability of the raw material and with the low process control on the marketed end product, especially concerning the freeze-thaw cycles that the meat often has to suffer with a worsening of its quality. In order to guarantee good quality of meat products the industry is looking for instrumental systems to assess and certify the product quality of this food supply chain.

In their paper, Banach *et al*. [7] showed that only 72 hours post-slaughter, a sample of bovine meat presented an impedance module (149 Ω), which practically coincided with the module of its resistive component (145 Ω) when a current with a frequency of 10 kHz was injected into that meat sample. From these latter data it can be argued that, when an electrical current with a high frequency circulates in the meat, the capacitive membrane-dependent component of the Z_m_ could be totally nullified and, at the same time, the impedance module reflects only its resistive component which could consent the expression of quality attributes of meat in the form of numerical values [7].

Starting from all the above considerations, the aim of this study was that of developing and testing a portable and cheaper device able to safely and easily assess the Z_m_ values of slaughtered beef muscles by injecting an electrical current with a high frequency, the preset value of which surely short-circuits the capacitive reactance of the cell membranes and thereby reduces the Z_m_ to just the intra- and extra-cellular resistive components [8].

The present work is available on preprints.org (https://www.preprints.org/manuscript/201905.0136/v1).

## Materials and methods

### The analog electrical model of the muscle

Fricke and Morse [9,10] have previously described an analogous electrical model which equates biological tissue components to passive electrical elements like resistors and capacitors connected in series and parallel.

**Fig.1:**
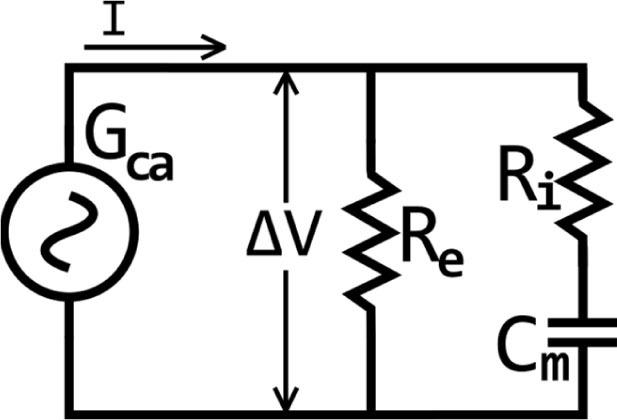
Analogous electrical model which equates biological tissue components to passive electrical elements. R_e_ is a resistor which mimics extra-cellular fluids together with some connective tissue elements and is parallel to R_i_ which mimics resistance of intracellular fluids and is serially connected to the capacitor C_m_ representing electrical charges separated by the cell membranes. When the alternate current generator G_ca_ is connected to the circuit at its ends, it generates a difference of electrical potential ∆V which induces the current I to flow in the two parallel branches of the circuit.

So, as is shown in Figure 1, a possible electrical model which could consent the expression of quality attributes of meat may consist of a resistor including extra-cellular fluids together with some connective tissue elements (R_e_) connected in parallel with the resistor including intra-cellular fluids (R_i_) which is in series with the membrane capacitors (C_m_) [7]. The meat's electrical impedance (Z_m_) can be calculated as in Equation (1), where j is the imaginary unity.

(1) $$Z_{m}=\frac{Z_{s}R_{e}}{Z_{s}+R_{e}}=\frac{\left[\left(R_{i}R_{e}\right)-\left(jX_{m}R_{e}\right)\right]}{\left[R_{i}+R_{e}-jX_{m}\right]}$$

Equation (2) expresses the X_m_ that is the capacitive reactance and f is the frequency of the injected current in the circuit. Equation (3) describes the Z_s_, series impedance, formed by R_i_ and X_m_.

(2) $$X_{m}=\frac{1}{2\pi f\;C_{m}}$$

(3) $$Z_{s}=R_{i}-jX_{m}$$

It is easy to deduce that as f progressively increases the X_m_ value tends towards 0, and Z_m_ tends to the expression of Equation (4), or rather, the meat's electrical impedance becomes dependent purely on changes in both intra- and extra-cellular resistive structures, which can be strongly conditioned by the maturation process of the meat [4] or, when occurring, on ice crystals between and within muscle fibers if the meat has been submitted to freezing/thawing processes [11,12,13].

(4) $$Z_{m}=\frac{R_{i}\cdot R_{e}}{R_{i}+R_{e}}$$

#### Instrumentation

On the basis of the analogous electrical model of Fricke and Morse [9] and to reach our goal, we applied a portable and single-frequency device with the capacity to easily assess measurements of the resistive components of the electrical impedance in the trimmed meat from beef carcasses, actuated at the production sites and with the possibility of sending bioimpedance signals to a remote control center. The afore-mentioned device is our own development, called Z_Meat_, consisting of three separate blocks: a sampling front-end, a command and control system and a data transmission system.

**Fig.2:**
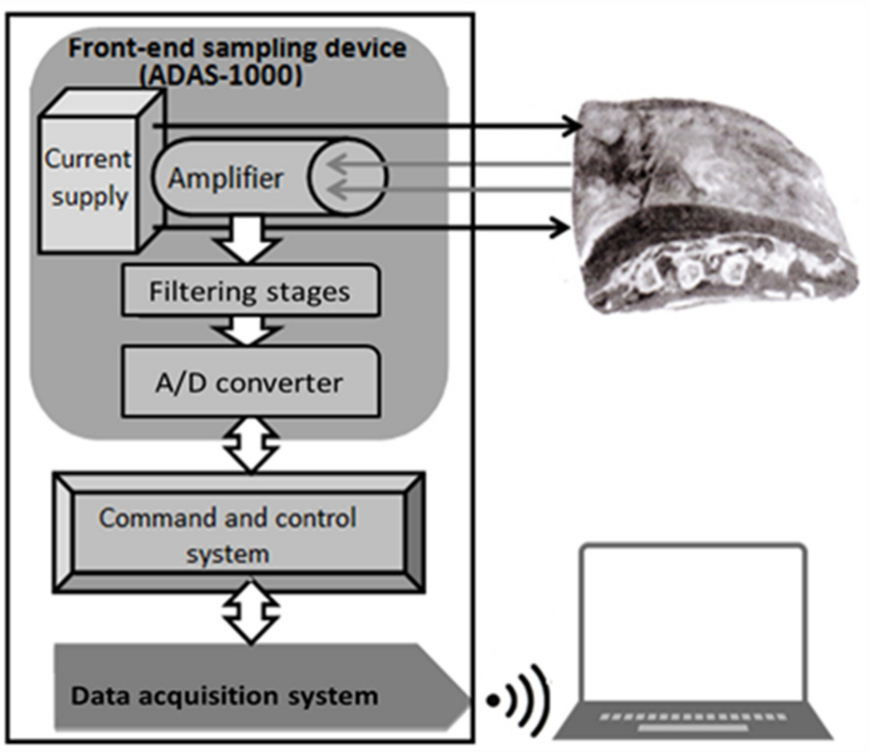
The block diagram is a schematic representation of the electrical components in the device Z_Meat_.

As is shown in Figure 2, the first block of the Z_Meat_ is the sampling front-end, which contains an alternate current supply that injects 1 mA at 65 kHz into the meat sample using two copper electrodes (black arrows) while two other electrodes connect the meat to the amplifier section (gray arrows). The front-end also contains a three-stage filtering section to ensure the elimination of frequencies outside the range of interest from the acquired signals. In the last stage of the front-end a digital conversion operation is performed by an appropriately calibrated 10 bit analog-to-digital converter. The core of the sampling front-end is the implemented and very cheap (about USD 200) ADAS-1000 evaluation board (Analog Devices, USA [14]). The command and control block supervises the sampling sessions by initializing and driving the ADAS-1000's operations and sends all the information to a controlling software operating on a personal computer by means of a data transmission system which contains a Bluetooth^©^ microchip fully supporting the radio frequency communication protocol, which enables a totally wireless operation mode of the Z_Meat_.

A chosen signal injected frequency of 65 kHz was used because other authors verified that at 10 kHz the values of impedance and resistance over a longissimus dorsi portion were practically the same [7]. So, a higher frequency such as 65 kHz guarantees that X_m_ has been eliminated. At the same time, the Z_Meat_ core is identical to another development of the authors dedicated to bioimpedance measurements [15,16,17,18,19].

#### Ethical approval

The conducted research is not related to either human or animal use.

#### Experimental procedures

Samples studied were obtained from four steers (half-breed Belgian blue cattle with Friesian) of about 16 months reared in a barn [20]. These were tested by the Z_Meat_. The considered carcass portions were longissimus dorsi muscles (LDm) of both sides corresponding to the VII–XI thoracic vertebrae.

**Fig.3:**
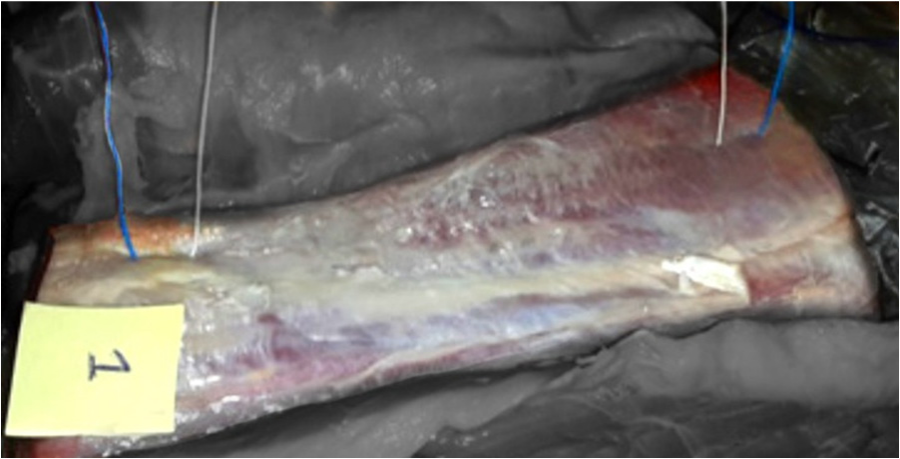
The picture shows the trimmed portion of a right longissimus dorsi muscle from one of the considered animals (285 kg in weight), which was freed from other muscular structures adjacent to it, and was oriented with its rostral head to the upper right. The two external wires are the current injection electrodes and the two internal wires are the electrodes to detect the voltage difference during test number 1 undertaken at the 2^nd^ day post-slaughter.

Trimmed LDm portions (see Figure 3) were 25 cm length with a rostral diameter of about 15 cm and a caudal diameter of about 10 cm, and each weighing from 6 to 8 kg. Each LDm portion was collected from a local abattoir 6 hours after slaughter and was packed in a plastic bag.

In these experiments, inspiration was drawn from the experimental protocols applied in previous experiments concerning the sequence of critical times to be applied just after slaughter LDm to obtain a good maturation [21]. On obtaining the samples from the local abattoir, both sides of LDm were immediately placed in a chilled room at 5 °C. The right sides of LDm stayed in the chilled room up to the 6^th^ day post-slaughter. The left sides of LDm, from the 3^rd^ until to 6^th^ day post-slaughter were frozen at −18 °C and then, at the 7^th^ day post-slaughter, these samples were returned to the initial chilled room at 5 °C for thawing.

#### Measurement protocol

**Fig.4:**
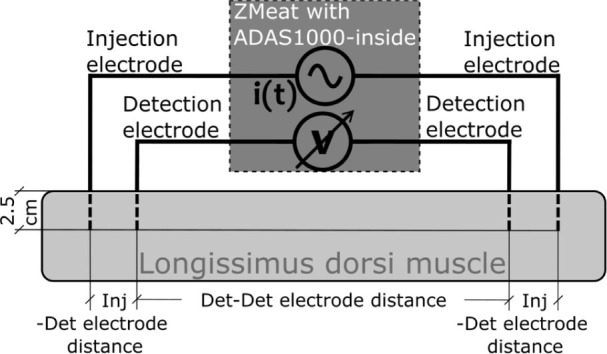
Schematic diagram of the Z_Meat_ measures of the beef samples of longissimus dorsi muscles.

Experiments for Z_m_ measurements of both LDm groups were undertaken in the chilling room with the following post-slaughter periodicity: 2^nd^ day (or thereabouts after the rigor period), 7^th^ day. So, in both groups of LDm each of the two Z_m_ measurements, i.e. the first as basal values and the second as post heat treatment values, were made at an identical time after the animals’ slaughtering. Figures 3 and 4 shows that the impedance measurements were carried out by utilizing the tetrapolar scheme [22], which uses two electrodes to inject a current flow I into the meat sample and two different electrodes to measure the voltage ΔV between these two electrodes and to deduce electric impedance by applying Ohm’s law (Equation (5)).

(5) $$Z_{m}=\frac{\Delta V}{I}$$

The four electrodes were annealed and cold-drawn copper wires with a diameter of 0.5 mm. Each electrode was inserted manually in the sample of the meat for a length of 2.5 cm.

Impedance measurements were made by inserting the four electrodes along the same line, ideally located along the muscle fibers and each pair of injection or detection electrodes was placed symmetrically with respect to the ideal point corresponding to one half of the muscle length. (see Figure 4).

From each meat sample 9 measurements of Z_m_ were taken and in each one of these, distances between the injection-detection (Inj-Det) pair of electrodes and the detection (Det-Det) pair of electrodes were modified as shown in table 1.

**Table 1:** Distance between electrodes.

Test number	Inj-Det electrodes^1^ [cm]	Det-Det electrodes^2^ [cm]
1	2.0	17.0
2	4.0	12.5
3	6.0	9.5
4	8.0	5.5
5	9.0	3.5
6	7.5	4.0
7	5.0	5.0
8	2.5	6.0
9	2.0	9.0

1 Distance refers to each pair of injection (Inj) and detection (Det) electrodes;

2 Distance refers to the two detection electrodes.

The procedure was carried out in this way to eliminate as much as possible any influences on the measured Z_m_ values due to differences in the relative positions of the electrodes. The time span between two consecutive measurements was 10 minutes.

Sampling tests were set for a minimum duration of 20 seconds each. The Z_Meat_ was configured to always operate at a sampling frequency of 2 kilosamples per second, recording a total of about 40,000 samples during each individual session.

#### Data analysis

Measured values of Z_m_ were parametrized as Ω per cm (Ω/cm) for scaling the differences between each measurement ‘Test Number’ (see Table 1). A similar data treatment was carried out by Gabriel *et al*. [23,24,25] even concerning different biological tissues where the materials were anisotropic and heterogeneous.

Measured values of parametrized Z_m_ from all four tested animals were subdivided into the following three groups: Z_mF_ containing impedance measurements from the left LDm frozen samples made at the 7^th^ day after slaughter; Z_mNF_ containing impedance measurements from the right LDm non-frozen samples made at the 7^th^ day after slaughter. Since no statistical difference was found between the two groups of the Z_m_ measured in both right and left LDm at the 2^nd^ day after slaughter, they were included in the same group; Z_mB_, which served as the baseline control of the two subsequent heat treatments.

Mean values ± SD were calculated for each data group and differences between them were statistically evaluated by the Student's t-test and, where appropriate, nonparametric rank tests were also applied. Differences among tested data were considered as significant for P<0.05.

Statistical tests were carried out utilizing commercially available software (MedCalc, Belgium).

## Results

Table 2 shows that 7 days after the animals’ slaughter the Z_mNF_ samples of LDm which aged 4 days at 5°C in the chilled room after the Z_mB_ measurements, reduced its value slowly (about −12%), even though significantly, with respect to that of the Z_mB_. On the contrary, the Z_mF_ samples of LDm which stayed 4 days after the Z_mB_ measurements at −18°C before thawing, showed a larger and significant reduction (about −39%) with respect to that of the Z_mB_.

**Table 2:** Mean impedance values among the tested beefs.

Z_mB_ [Ω/cm]			Z_mNF_ [Ω/cm]			Z_mF_ [Ω/cm]		
N	Mean	±SD	N	Mean	±SD	N	Mean	±SD
72	6.24	0.14	36	5.51*	0.35	36	3.81*^†^	1.41

Mean ± SD of the electrical impedance values of the slaughtered meat in the base condition (Z_mB_), not frozen (Z_mNF_) and frozen and thawed (Z_mF_) conditions. N: number of measurements;

* P<0.0001 with respect to Z_mB_;

† P<0.0001 with respect to Z_mNF_.

**Fig.5:**
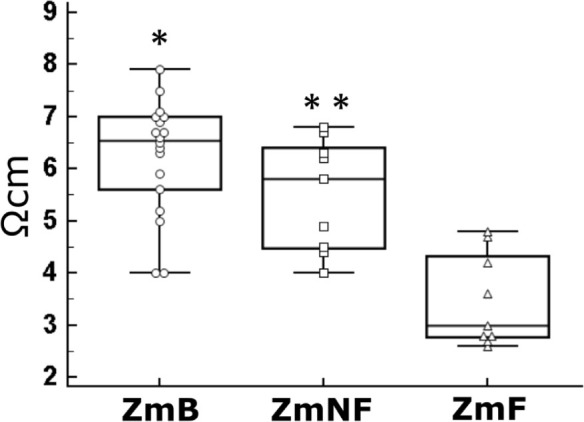
The experimental data obtained from one of the four studied animals and concerning the electrical impedance values (Z_m_) of the slaughtered meat in the base condition (Z_mB_), non-frozen (Z_mNF_) and frozen and thawed (Z_mF_) conditions, are represented as box and whiskers diagrams with markers (empty symbols) that reveal the distribution of each measurement. With respect to Z_mF_: (*) P = 0.004; (**) P = 0.004.

To highlight the different behavior of Z_m_ when LDm were submitted or not to a freezing treatment, Figure 5 shows a graphic representation of a typical behavior of the impedance in the longissimus dorsi muscle samples concerning one of the tested animals. Due to the asymmetric distribution of the data, the visualization of the assessed values of the specific impedance was made by means of box and whiskers plots [26,27] in which we have also included the spread of data. Figure 5 shows a practically complete overlap between the third quartile (Q3) of the Z_mB_ box and the first quartile (Q1) of the Z_mNF_ box, thus their respective median lines are very close (Z_mB_ = 6.6 Ω/cm and Z_mNF_ = 5.8 Ω/cm) with no statistically significant difference between them.

The same Figure 5 clearly shows that the box of Z_mB_ data is placed totally above the box of Z_mF_ data, and the median value of this latter group is lower than half that of the base group of data. For this reason, statistical comparison between these two groups of data showed high significant difference. When comparing both the Z_mNF_ and the Z_mF_ box, it appears that the Z_mNF_ box is also placed above the Z_mF_ one, and the median of non-frozen group data is 1.9 times higher than that of the frozen one. Also, this difference between Z_m_ values turned out to be highly significant.

## Discussion

Results arising from these experimental measures of the meat's electrical impedance reasonably consent us to consider 65 kHz of frequency for the applied signal for Z_m_ monitoring, i.e. that released from the Z_Meat_, as a reliable frequency for giving information concerning the ageing-dependent maturation of bovine meat.

In fact, it must be taken into account that ageing-dependent maturation of meat is due to a progressive disruption of the composite network of myofibres with their surrounding envelopes of connective tissue. This leads to an irreversible morpho-functional alteration of the cytoskeleton and cell membranes of myocytes [2] with a progressive loss of the histological anisotrophy of the muscle tissue. This loss of the morphological anisotropy induces a parallel reduction in the muscle's electrical anisotropy [28] which is revealed by a progressive falling of its electrical impedance [21] which coincides with its real, i.e. resistive, component.

Considering that in its hardware, the Z_Meat_ implements a current generator which delivers a current flow into muscle tissue with a frequency of 65 kHz, or a frequency which is higher than 10 kHz, which has been found to nullify the muscle capacitive reactance [7], then it is reasonable to assume that our Z_Meat_ measures the resistive components as described in the muscle's electrical analog model [9], i.e. this Z_m_ monitor could give information on the ageing-dependent maturation of meat in the form of numerical values.

Paying attention to the results from these experiments, data indicate that, at the 7^th^ day from slaughter, a portion of the beef longissimus dorsi muscle frozen at −18° C in the previous 4 days, when thawed, showed a Z_m_ value which was almost half that of the value assessed in the 2^nd^ day post-slaughter, before its freezing. These results were in good agreement with those previously found by Banach *et al*. [7] that, in a bovine meat sample which was frozen for three months at −24° C, when thawed, showed a Z_m_ which was 3.6-fold lower than that measured just after slaughter.

That described above, from both ours and Banach *et al*.'s experiments, is supported by robust histological evidence that highlights the quick and dramatic damage done to the sophisticated fibrillar organization of muscles by the very rapid formation of ice crystals when the meat has been frozen.

In fact, in the mile-stone paper by Rahelić and Puać [29] it was clearly demonstrated that when samples of beef longissimus dorsi muscle were frozen at temperatures between −10 °C and −22 °C ice crystals formed both between muscle cells and inside these cells. In fact, these authors showed that histological changes in muscles frozen at −10 °C presented in cross-section groups of several tens of fibers. These groups of fibers were separated by large intercellular spaces due to ice crystal formation. In these groups, muscle fibers were attached to one another and individual groups were linked by one or two rows of fibers. Otherwise, when the muscle samples were frozen at −22 °C the fiber groups were smaller with respect to those frozen at −10 °C and were separated by smaller interspaces. However, there are frequent gaps in the middle of fibers due to ice crystals formed inside the cells. This study concluded that damage is most severe in the muscles frozen at −22 °C. To justify this claim they proposed that opposing pressures through cell membranes are created from outside the cell by large ice crystals formed intercellularly and from inside the cell by pressures created by the formation of ice crystals here too. As a result of such opposing pressures, the tearing of the fibers will be greater.

More recently, Egelandsdal *et al*. [30] utilized the method of the Cryo-SEM (cryo-scanning electron microscopy) in samples of pork loin muscle frozen for 3 days at −25°C. After 14 hours of thawing the meat samples up to 3°C, the Cryo-SEM images at 500 × magnification identified large cavities (more than 1 μm) in the muscle samples indicating ice-crystal formation due to freeze damage [31]. In these muscle samples the regular arrangement of fibers interspersed with perimysial connective tissue was essentially lost, and typical anatomic structures of meat became non-identifiable since extensive cavity formation was observed in the frozen meat.

Even though in the present experiment no histological examination of the meat samples was carried out either at the beginning or at the end of the two experimental treatments, on the basis of the previous experimental results as reported above, it is reasonable to assume that in our bovine longissimus dorsi muscle samples that were frozen for 4 days at −18 °C, the histological structure underwent dramatic changes due to formation of ice crystals both outside and inside the muscle fibers.

These occurrences are even more supported by a recent histological study of samples of both longissimus lomborum and semitendinosus beef muscles, which were frozen at −20 °C for 3 days after slaughter [32]. Observing muscle micro slices under the light microscope the authors of these experiments found the presence of ice crystal formation in more elongated shapes and widely distributed patterns. Moreover, in the same experiments, muscle samples frozen at −20 °C for 3 weeks and after thawing, showed more gaps between muscle fibers compared to the ones that underwent maturation at 2 °C for 2 weeks. This occurrence could further justify the disruption of the histological anisotropy, as described by Rahelić and Puać [30], which could happen when muscle samples are frozen at low temperatures around −18 °C, i.e. what could happen to our left side longissimus dorsi muscles of beef samples.

This evidence would lead us to expect a remarkably better electrolytic conductance in frozen meat, in comparison to the non-frozen one, which is linked with the rapid and profound change in its anisotropic structure, due to the formation of ice crystals, i.e. an alteration of insulating properties of cellular membranes allowing a great mobility of ions could happen [11,33]. The degree of this change depends on the rate of the freezing process and the amount of water inside the structure of muscle tissue [34]. In the present experiment we also found that, as expected, after 7 days post-slaughter the Z_m_ of the sample muscles which had been frozen in the previous 4 days and then thawed were almost a half that of the only-aged one. This latter result reinforces the validity of this 65 kHz Z_Meat_ monitor to also discriminate between fresh and frozen-thawed beef meat.

## Conclusions

These experimental results reasonably demonstrate that our Z_Meat_ prototype applied to Z_m_ monitoring is a reliable device to assess resistive components of the electrical impedance in trimmed longissimus dorsi muscle of slaughtered bovine since impedance values assessed by it behaved in agreement with those of previous experiments concerning the same matter.

The use of a custom designed device with a dedicated control software allowed the development of a low cost, compact, portable tool able to automatically and wirelessly perform all the measurements of the resistive components of the electrical impedance in the trimmed meat in real-time. So, this device might meet the needs of the bovine meat industry especially when it is necessary to rapidly check, in the place of production or storage, if such meat is fresh or previously frozen.

## Study Limitations

A possible criticism for this manuscript could be drawn due to the lack of histological evaluations of our meat samples both at the beginning and the end of the two experimental treatments. However, many previous experiments [29,30,31,32] have already investigated in great depth into the ultra-structural changes that animal elongated muscles undergo when frozen at temperatures close to that used in this experiment, and the results of these previous experiments can reasonably be used as a reference of the possible histological damage that also occurred in our samples of bovine longissimus dorsi muscles. Nevertheless, we are looking for a skilled histological laboratory which could be capable of providing information concerning the ultrastructural changes of beef muscle samples to be compared with our Z_Meat_ data.

Another possible criticism could be drawn due to the relatively small number of animals studied. However, it is quite possible that calves coming from the same breeding and of the same age and weight would also present very similar values of morphological and functional parameters due to the high affinity in genetic architecture of livestock breeds [20]. Nevertheless, the data reported in this paper are based on a pilot study to be followed as soon as possible by a confirmatory study based on a larger sample of tested animals.
